# A Flexible Kenics Mixer for Applications in Liquid Chromatography

**DOI:** 10.3390/mi14071373

**Published:** 2023-07-04

**Authors:** Prachet Dsk, Petru S. Fodor, Chandrasekhar R. Kothapalli

**Affiliations:** 1 Department of Aerospace Engineering, Indian Institute of Technology Bombay, Powai, Mumbai 400076, India; 2 Department of Physics, Cleveland State University, 2121 Euclid Avenue, Cleveland, OH 44236, USA; 3 Department of Chemical and Biomedical Engineering, 2121 Euclid Avenue, Cleveland, OH 44236, USA

**Keywords:** Kenics mixer, micromixer, microfluidics, flexible tubing, liquid chromatography

## Abstract

Miniaturization of liquid chromatography could help enhance sensitivity, reduce solvent usage, and detect small quantities of peptides. However, it demands better sample homogenization of the mobile phase. We here developed a mixer design based on the inline Kenics geometry, consisting of a periodic arrangement of twisted blades placed inside a cylindrical capillary that repeatedly cut and stack fluid elements to achieve rapid mixing in laminar flow regimes. The mixer design was optimized with respect to the twist angle and aspect ratio of the mixing units to achieve complete mixing at minimum pressure load cost. Results suggest that for optimal designs, for a mixer volume of ~70 μL, complete mixing is achieved within a distance smaller than 4 cm for a broad set of flow rate conditions ranging from 75 μL·min^−1^ to 7.5 mL·min^−1^. A salient feature that we introduce and test for the first time is the physical flexibility of the cylindrical capillary. The performance of the design remained robust when the mixing section was not rigid and bent in different topologies, as well as when changing the chemical composition of the mobile phase used.

## 1. Introduction

Liquid chromatography (LC) systems have become a mainstay in analytical chemistry, being widely used to separate, identify, and quantify with high precision components in a mixture [1,2], with applications in pharmaceuticals analysis [3], forensics [4], environmental [5] and food analysis [6], to name just a few. Typical systems rely on pumps to pass pressurized solvents (also referred to as the mobile phases) in which the sample mixture of interest is injected through a column filled with packed adsorbent particles. The individual ingredients from the sample mixture are then separated based on their varying degrees of interaction with the adsorbent particles [7]. One of the critical characteristics needed to achieve high detection performance is for the fluids used in the mobile phase to be well mixed before reaching the column [8]. Deviations from homogeneity result in noisy wave patterns in the baseline and detection sensitivity reduction through band broadening and reduced reproducibility of retention times [8]. For less demanding applications, sufficient mixing and smoothing of the irregularities in the composition can be achieved simply from the collision of the mobile phase fluid streams at their convergence point, typically a T- or Y-junction, and from the turbulent flows that result from the interaction of the fluids with the various turns and valves within the system [9,10]. For more demanding applications and operation at lower flow rates, optimal operation needs better homogenization which requires the placement of performant mixers after the convergence point of the mobile phases and before the sample injection [11], and sometimes even between the injector and the column [8]. The use of mixers can be effective in preventing flow instabilities that result from mismatches between the physical properties of the mobile phases, such as viscous fingering that occurs when a less viscous solvent (e.g., acetonitrile) is injected into a more viscous fluid (e.g., water) [12].

Although actively mixed chambers employing magnetic or mechanical stirrers have been used in LC, most mixers employed for these types of applications are passive ones that do not require external power and do not have moving parts [11]. This presents obvious advantages when mixing and matching pump and column systems, as well as minimizing the volume added to the system. Performant passive mixing systems, some available commercially, have been developed for these applications. Examples include the SMX mixer [13], where periodic x-shaped crossbars are placed in the path of the flow generating folding and stretching of the fluid elements, and 3D printed serpentine mixers [14] that rely on mixing promoted by cross-sectional flows induced by the centrifugal forces experienced by the fluid as it is forced to travel along curved channels. It must be noted that in the above systems, the mixing efficiency strongly depends on the fluid speed, with the homogenization quality being sufficiently high when higher flow rates and larger volumes are involved [15]. For situations where the mixing of small volumes is required at a wider range of flow rates [16,17], the above solutions are not always suitable. To this end, in recent years, new mixer designs had to be developed for the low volume/slow flow rates setup, based on microfluidic technologies that can achieve good mixing even in the low Reynolds number regimes (1 < *Re* < 100), where the flow is laminar. These mixers, when applied in the context of LC, employ mixing methodologies developed for microreactors and micro-assays. Examples include using ridge-groove systems, or Dean flows in spiral channels to generate cross-sectional flows, as well as multilayered channels that lead to splitting-and-recombination of the fluid flows [18]. At low Reynolds numbers where the flow is laminar, these approaches overcome the challenges associated with achieving good mixing quality in a diffusion-limited system where turbulence is absent. Their intricate design and complex geometries could be optimized with advanced numerical calculations, while their implementation could be practically achieved with fabrication methodologies such as 3D printers.

One particularly successful mixing strategy that has been employed for systems in the laminar flow regime and has desirable characteristics for LC applications, such as high efficiency and performance, low dead volume, low pressure drop relative to other designs, promotion of plug flows, and easy flushing after use, is the so-called Kenics geometry [19]. This inline design involves the use of helically twisted plates inserted in cylindrical pipes that divide the system into two twisted semicircular channels. The helical twisted plates are placed along the fluid flow in a periodic arrangement, with the leading edge of each plate being rotated normally to the trailing edge of the previous plate. The resulting repeated splitting, stretching, and stacking of the fluid volumes mimics the baker’s transformation [20], leading to the formation of striations within the fluid volume and effective mixing. To date, this mixer technology has proved to be versatile, owing to its low-energy mixing capabilities, with many industrial applications ranging from agriculture, food technology, chemical processing, pharmaceuticals development, minerals processing, waste treatment, and mixing of polymers, plastics, paints, and resins. Their periodic geometry lends itself to optimization in terms of geometrical parameters (diameter, length, blade twist angle, and mixing unit aspect ratio) and flow rates using numerical simulations that correlate well with experimental results. The structural parameters in studies using Kenics mixers typically ranged as follows: twist angles from 90–180°, pipe diameter from 1.2 mm–5 cm, 1 ≤ *Re* ≤ 1000, and the number of elements from 3–25, depending on the application or flow rates used.

Inspired by the above work, in this current study, we are proposing a mixer for LC applications based on the Kenics geometry. Distinct from previous industrial applications of this design, in which barrel diameters of 50 mm or larger are not unusual, the size of the mixer is scaled down appropriately for LC systems, with an inner diameter (I.D.) of 1.6 mm (corresponding to 1/16″ I.D. Peek capillary tubing). An extensive computational optimization of the mixer geometry is performed to identify the geometrical parameters of the mixing units conducive for optimal mixing on these scales in terms of performance and energy cost. Optimal mixer designs with a volume ~70 μL are found to achieve complete mixing for a broad set of flow rate conditions ranging from 75 μL·min^−1^ to 7.5 mL·min^−1^, and across typical mobile phases used in LC, such as water, acetonitrile, and methanol. The optimized design is accessible to rapid prototyping, where the core functional unit of the mixer, i.e., the mixing blade unit, can be 3D printed and simply inserted into the connecting capillaries. For instance, an Objet 350 Connex3 printer (Stratasys, Ltd., Rehovot, Israel) with a resolution of 600 × 600 × 1600 dpi (X-Y-Z-axes respectively) and accuracy of around 20–85 µm could be used to 3D print such small features of our proposed mixer designs. Thus, the addition of this mixer to the LC system can be done without adding additional volume while also bringing forward the intriguing possibilities of having flexible connections to the column, as well as further increasing the mixing quality by taking advantage of the cross-sectional flows that developed when fluids are forced to move along curved channels [21,22]. To this end, the performance of various topologies with different channel curvatures is analyzed. The data confirm that the optimized design performs very well with ≥95% mixing performance, with slightly better results for geometries with a smaller radius of curvature.

## 2. Mixer Geometrical Design

**Figure 1:**
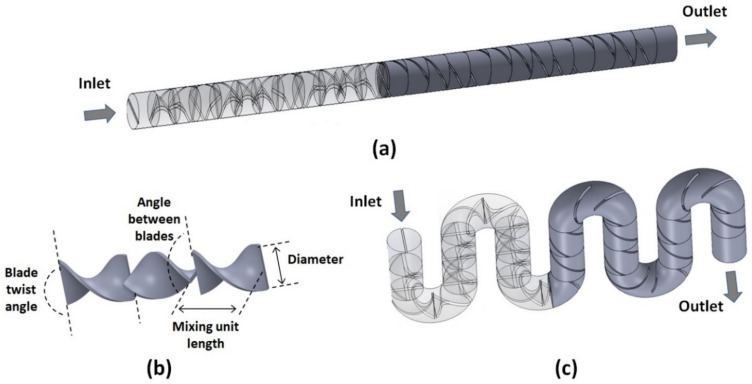
(**a**) Wireframe (left) and solid (right) projections of a straight Kenics mixer structure with 24 mixing units. The wireframe view shows the structure of the mixing elements; (**b**) Typical geometrical parameters of the mixing screw design; and (**c**) Wireframe (left) and solid (right) projections of the curved Kenics mixer with two mixing units within the curvature.

The structure of the flexible Kenics micromixers designed here consists of a circular channel with a constant inner diameter of *D* = 1.6 mm, with a periodic arrangement of mixing elements placed along the fluid flow (Figure 1a). Each mixing element consists of a helical rectangular plate twisted along the length of the element by a fixed angle, as measured between the trailing and leading edges of the plate (Figure 1b). In the performance optimization study relative to the twist angle of the blade, values between 90° and 180° are explored. The length *L* of the mixing unit is defined based on its desired aspect ratio *AR* = *L*/*D*, which is varied from 0.75 to 2.5 when optimizing designs relative to this geometrical parameter. In all the studies presented, the thickness of the blades is set to 10% of the channel diameter. Each subsequent mixing unit is rotated so that its trailing edge is rotated by 90° with respect to the leading edge of the previous unit. Thus, this geometry splits the fluid flow at the entrance of each mixing unit leading to the stacking of the fluid elements. Additionally, the rotation of the mixing elements relative to each other forces changes in the rotation of the flow that leads to the stretching of the fluid elements, which can be conducive to promoting higher mixing efficiency [23,24].

Most of the studies presented in this work are for systems that contain curved sections (Figure 1c), as the mixers presented are assumed to be flexible and thus can assume serpentine topologies. For these cases, for the straight sections of the channel, the mixing units remain as described above. As for the curved sections, each one subtends an angle equal to 180°/*n* where *n* is the number of mixing units considered within the curvature. The curved sections are designed under the constraints that the arc circle corresponding to their central line is fixed at the original straight mixing unit length, *L*, and that the cross-section of the mixing unit remains unchanged.

**Figure 2:**
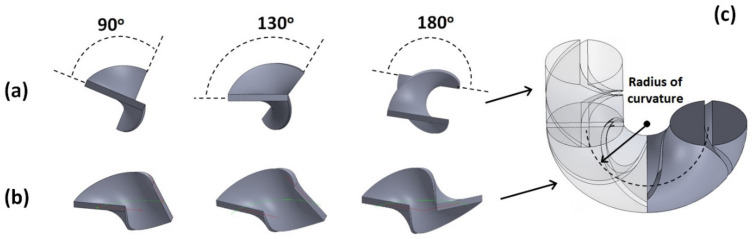
Structure of the mixing screw elements with different twist angles for (**a**) straight sections; and (**b**) mapped on curved sections; (**c**) Illustrative section of a Kenics mixer with both straight and curved sections (for the case illustrated, the blade twist angle = 180°).

Consequently, the points defining the mixing blade are mapped onto the curved channel, with points on the outer periphery being stretched apart while points on the inner periphery being brought closer together (Figure 2a,b), with the angles that the trailing and leading edges form with the longitudinal plane staying the same as for a straight section (Figure 2c). The relative rotation between subsequent mixing units is also kept the same, i.e., 90°, as measured between the trailing edge of a mixing unit and the leading edge of the previous one.

## 3. Numerical Modeling

To evaluate the mixing quality within these systems, the flow and concentration-diffusion equations are numerically solved. First, the flow fields within the geometry are obtained by solving the Navier–Stokes equations (momentum and continuity) under the assumption that the fluid is incompressible and Newtonian under a steady-state pressure-driven flow:

(1) $$\rho \left[\frac{\partial u}{\partial t}+\left(u\cdot \nabla \right)u\right]=-\nabla p+\eta \nabla^{2}u$$

(2) ∇·u=0

where ***u*** [m·s^−1^] is the velocity vector, *ρ* [kg·m^−3^] is the fluid density, *η* [kg·m^−1^·s^−1^] is the fluid viscosity, *t* [s] is the time, and *p* [Pa] is the pressure. Depending on the fluids employed for the mobile phase, the values for the density and viscosity are set as: (i) *ρ* = 0.998 × 10^3^ kg·m^−3^ and *η* = 1 × 10^−3^ kg·m^−1^·s^−1^ for water at room temperature; (ii) *ρ* = 0.786 × 10^3^ kg·m^−3^ and *η* = 0.343 × 10^−3^ kg·m^−1^·s^−1^ for acetonitrile; and (iii) *ρ* = 0.791 × 10^3^ kg·m^−3^ and *η* = 0.575 × 10^−3^ kg·m^−1^·s^−1^ for methanol. The mean velocities at the inlet are changed from 0.627 × 10^−3^ m·s^−1^ to 62.7 × 10^−3^ m·s^−1^ (corresponding to Reynolds numbers *Re* = 1 to 100) to achieve flow rates ranging from 75 μL·min^−1^ to 7.5 mL·min^−1^. No-slip boundary conditions are set for the walls of the micromixers, and the flow field equations are solved using a generalized minimal residual method (GMRES) iterative solver with a geometrical multigrid pre-conditioner. A free tetrahedral mesh is used for the entire channel with a typical mesh density of 4.35 × 10^4^ mm^−3^. Preliminary studies on optimizing the mesh independence are provided in Supplementary Data Figure S1. For all the simulations described in this work, we used the computational package COMSOL Multiphysics (Version 5.1, COMSOL, Inc., Burlington, MA, USA) and its Computational Fluid Dynamics/Chemical Engineering module.

The flow fields determined in the previous step are then used to determine the distribution of the concentration *c* [mol·m^−3^] of a tracer throughout the volume of the mixer by solving the concentration-diffusion equation:

(3) $$\frac{\partial c}{\partial t}=D\nabla^{2}c-u\cdot \nabla c$$

where *D* [m^2^·s^−1^] is the diffusion coefficient.

The diffusion coefficient is set to the corresponding room temperature diffusion constant specific to the fluids used, i.e., (i) *D* = 1.0 × 10^−9^ m^2^·s^−1^ for water; (ii) *D* = 3.2 × 10^−9^ m^2^·s^−1^ for acetonitrile; and (iii) *D* = 2.3 × 10^−9^ m^2^·s^−1^ for methanol [25]. For the boundary condition for the concentration inflow, one half of the inlet is set at an entry concentration of 1 mol·m^−3^, while the other is set to 0 mol·m^−3^. Similar numerical methods as described for the flow equations above are used, with the exception that they are mapped onto a much finer grid with a mesh density of 2.7 × 10^5^ mm^−3^ to avoid numerical errors arising from the smaller scales associated with diffusional mass transport. These numerical models have been previously validated against experimental data in similar laminar systems containing curved sections and complex flow-generating structures [26,27], with good accuracy in terms of mixing quality assessment and concentration maps rendering.

## 4. Results and Discussion

**Figure 3:**
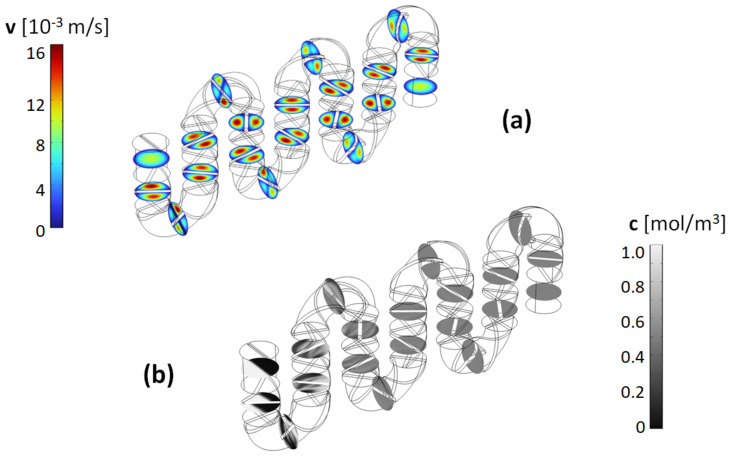
(**a**) Transversal flow field profiles and (**b**) concentration cross–sections for a channel with 130° blade twist angle and aspect ratio *AR* = 1 for the mixing elements, operated at *Re* = 1 (mobile phase fluid = acetonitrile).

Typical results for the flow field and concentration distribution as the fluid moves through the channels are shown in Figure 3. This geometry is representative of the ones used in this study, with mixing elements present in both the straight and curved sections of the channels. Although most of the optimization study is done for the topology of serpentine channels with two mixing units within the curvature, as discussed later, the mixing quality in this type of mixer is found to be very robust against changes in the topology of the channels. The velocity magnitude cross-sectional profiles (Figure 3a) show the splitting of the flow elements as they enter each mixing unit. As observed in the concentration profiles (Figure 3b), an immediate effect of the split-recombination sequence is the formation of striations within the cross-section of the flow, with fluid elements originally in different parts of the cross-section being interlaced. This is a direct consequence of neighboring blades being rotated perpendicular to each other. Moreover, it is also apparent that the twist angle of the helical blades imparts a rotational motion to the fluid elements as the orientation of the striations changes as the fluid moves along the channel. This should increase the mixing efficiency, as aside from being split and stacked, the fluid elements also experience sequences of stretching and folding.

The stretching of the fluid elements is further compounded in the curved section of the mixer. As observed in the velocity profiles, while those tend to be symmetric in the two halves of the channel defined by the mixing blades within the straight sections, that is not the case in the curved sections, where the symmetry is broken due to the centrifugal forces present. In these regions, the rotational flows resulting from the fluid being forced to move along the helical blades overlap with the cross-sectional Dean flows associated with the fluid motion being confined in a curved channel [21].

### 4.1. Blade Twist Angle Dependence

**Figure 4:**
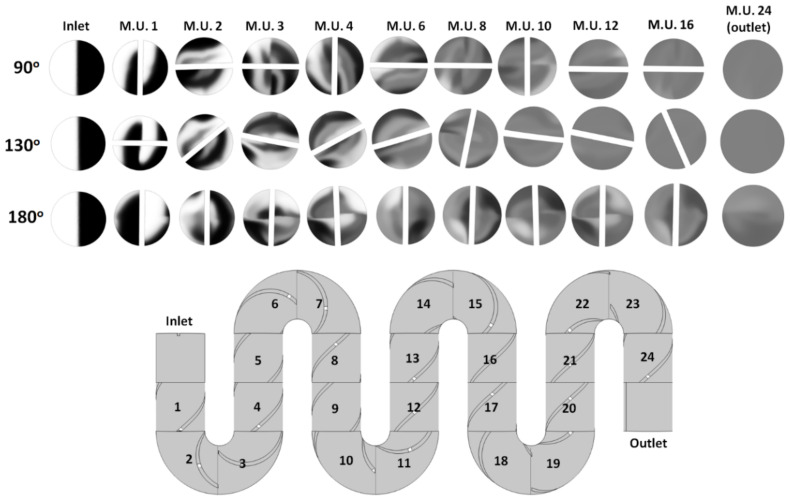
(**top**) Concentration profiles along the channel for designs with various twist angles (*Re* = 10; mobile phase fluid = water; aspect ratio *AR* = 1); (**bottom**) Side view to the channel showing the position (index) of individual mixing units (M.U.).

To observe the evolution of the mixing in the channel and identify optimal geometrical parameters, closeup images of the cross-sections of the concentration profiles are taken along the channel. The mixer topology used in the optimization study has 24 mixing units integrated into it, and the concentration profiles are taken after each mixing unit (M.U.) labeled based on their position along the channel, with M.U. 1 being the first mixing unit after the inlet and M.U. 24 being the last mixing unit just before the outlet. In previous studies with industrial Kenics mixers, it was found that one of the primary geometrical parameters that affects the mixing quality is the twist angle of the helical blade [28]. To this end, in our studies, we have explored geometries with blade twist angles ranging from 90° to 180° in steps of 10°. As observed in Figure 4, the concentration profiles for designs with different angles share similarities with increased striations as the fluid moves along the channel and consequently increased homogeneity in the concentration profiles. It has to be noted, though that there are also distinct qualitative differences between designs based on different angles in terms of how quickly the mixing occurs. For example, for mixers with a 180° twist angle, the progression toward homogeneity is quite slow. An analysis of the concentration profiles, in this case, indicates that while the blades induce fluid rotation, for this particular situation, the rotation is such that the fluid elements on opposite sides of the channel simply switch positions. Thus, when entering the next mixing unit, little striation actually occurs, as regions with similar initial concentrations remain connected. A similar effect, albeit less pronounced, is observed for the 90° twist angle design, where the fluid has to pass through multiple mixing units before homogenization occurs.

Previous studies have identified optimal angles from 110° to 140° [29,30], depending on the application or flow rates used. Here, we obtained a flat maximum within the same region, with the difference that beyond about 130°, the mixing quality starts deteriorating notably. This difference could be attributed to the fact that previous studies were for systems that were quite larger in diameter and were operated at different flow rates. Our current design is being optimized for the operating conditions relevant for LC applications.

On the other hand, for intermediate angles, where the striations generated in one mixing unit meet the next mixing unit at oblique angles, the homogenization of the concentration profiles is accomplished in noticeably fewer mixing units. To identify an optimized design in terms of the blade twist angle, the information captured by the concentration profiles can be used to define a quantitative mixing measure. One such measure is based on evaluating the variance in the concentration *c* relative to the mean concentration $\bar{c}$ [31,32], by calculating the degree of mixing using a mixing index *M* defined as follows:

(4) $$M=1-\frac{1}{\bar{c}}\sqrt{\frac{\sum_{i=1}^{N}{\left(c_{i}-\bar{c}\right)}^{2}}{N}}$$

where *N* is the number of mesh elements across each concentration cross-section, and *c_i_* is the concentration value at each element. For our inlet boundary condition where half of the flow is at a concentration of 1 mol·m^−3^, while the other is set to 0 mol·m^−3^, the mean concentration in the above Equation (4) will be $\bar{c}$ = 0.5 mol·m^−3^. The mixing performance measure thus defined will take values between 0 and 1, with 0 corresponding to full segregation while 1 corresponds to 100% mixing, respectively.

**Figure 5:**
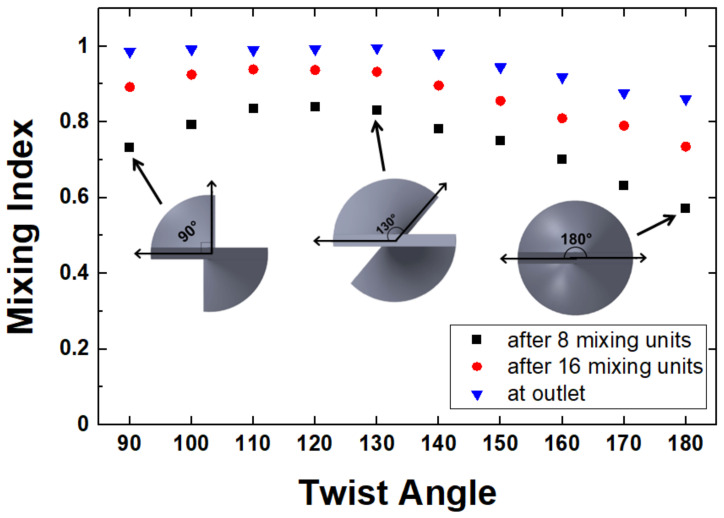
Mixing index dependence on the twist angle of the mixing unit at different positions along the channels. Insets show the corresponding geometry of the mixing screw (*Re* = 10; mobile phase fluid = water).

The evaluation of the mixing index is done directly in COMSOL by numerically integrating the cross-sectional concentration profiles corresponding to different positions along the channel. Figure 5 summarizes the results for designs with different mixing unit blade twist angles. The mixing index is quantified through 8 mixing units (a third of the way through the channel), through 16 mixing units (two-thirds of the way), and at the outlet, i.e., after all the 24 mixing units, respectively. Consistent with the qualitative observation of the level of homogeneity in the concentration images, the performance of the designs is noticeably affected by the twist angle chosen for the design. The mixing is maximized for angles between 110° and 130° for all positions along the channel while decreasing towards smaller and larger angles. After 24 mixing units, corresponding to a mixer volume of 67.4 μL and a length of 3.84 cm, for the 130° degrees design, the mixing is essentially complete with a mixing quality measure of ~99%.

### 4.2. Aspect Ratio Dependence

**Figure 6:**
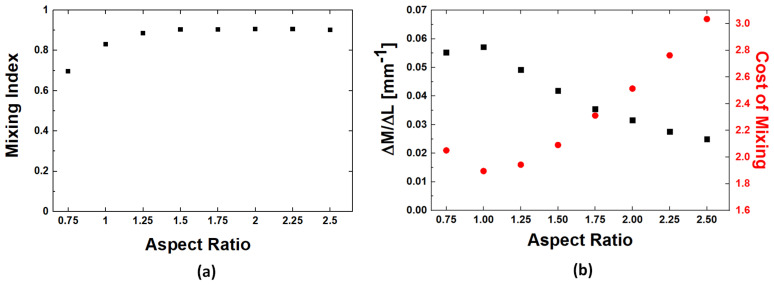
(**a**) Aspect ratio dependence of the mixing index; and (**b**) Aspect ratio dependence of the mixing index per unit length and the cost of mixing, respectively (*Re* = 10; mobile phase fluid = water; blade twist angle = 130°).

One other geometrical parameter that can influence the quality of mixing in Kenics type mixers is the aspect ratio of the mixing unit, i.e., the ratio (*AR* = *L*/*D*) of the length per diameter of the base unit used [33]. To capture the effect of this geometrical parameter, designs with a fixed blade twist angle of 130° and a diameter *D* = 1.6 mm but different mixing unit lengths corresponding to aspect ratios from 0.75 to 2.5 are analyzed. The mixing index is evaluated after 8 mixing units for mixer topologies as those shown in Figure 4. The results (Figure 6a) show an increase in the mixing quality as the length of the mixing units is increased. This increase in the mixing index for larger aspect ratios is not unexpected as larger *AR* values are also associated with longer residence times within the mixer and, thus, longer times for the components to diffuse. Thus, to better understand the effect of the aspect ratio on the mixer performance, one must account for the increase in the total length of the mixers when larger aspect ratios are used. To this end, we use two ways to normalize the mixing quantification. One relies simply on calculating the mixing increase per unit length Δ*M*/Δ*L*. The other measure calculates the cost of mixing (*COM*) by accounting for the pressure differential Δ*p* needed to push fluids through the mixer [31,34]:

(5) $$COM=\frac{\frac{\Delta p}{\rho u_{mean}^{2}}}{M\times 100}$$

where *ρ* is the density of the fluid and *u_mean_* is the mean velocity at the inlet of the system. The two measures, i.e., Δ*M*/Δ*L* and *COM*, are evaluated for the section of the mixer involving the first 8 mixing units (Figure 6b). Both measures show a clearly developed optimum value for the aspect ratio of *AR* = 1, where the mixing increase per unit length is maximized at a minimum energetic cost. Below this value, presumably, the length of the mixing blade does not impart sufficient rotation to the fluid elements, while for longer blade structures, the desired effect is already achieved with the extra length, only adding increased pressure differential requirements without improving the mixing performance.

### 4.3. Operations Conditions Dependence

**Figure 7:**
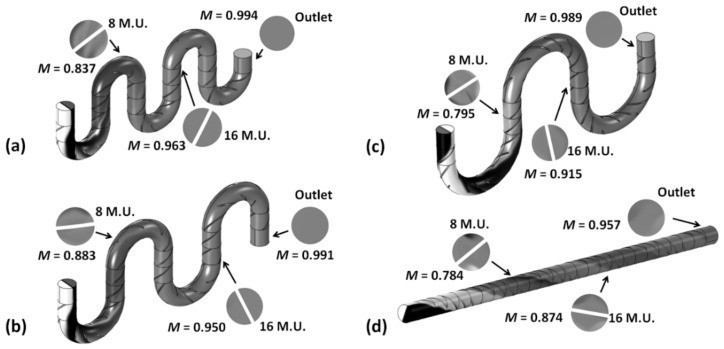
Evolution of concentration and mixing index along equal length Kenics channels with different numbers of mixing units (M.U.) within the curvature: (**a**) 3 units; (**b**) 4 units; (**c**) 6 units; and (**d**) straight channel (blade twist angle = 130°, aspect ratio *AR* = 1, mobile phase fluid = water, *Re* = 10).

The optimized structure identified from the above geometrical parametrization, i.e., blade twist angle ~130° and aspect ratio for the mixing unit *AR* = 1, has been tested under a broad range of operations conditions. One of the variables considered is the actual topology of the channel. Since the mixing system is assumed to be flexible, with the set of periodic mixing blades simply inserted into a flexible connecting capillary tube, it is conceivable that in operation, the mixer will assume various topologies, as defined by the number of mixing elements within the curvature of the microchannel (Figure 7).

The mixing index calculated at different positions along the mixer after 8 mixing units, after 16 mixing units, and at the outlet (i.e., after 24 mixing units) shows limited variations between topologies (Figure 7). Cases with sharper turns, i.e., fewer mixing units within the curvature, show slightly better mixing performance at all locations along the channels. This is consistent with the fact that when curvatures are present in the system the fluid rotations and splitting imparted by the helical blades are compounded by the development of Dean flows associated with the fluids being forced to follow the curved channel and experience centrifugal forces. Nevertheless, it must be noted that for all topologies, at the outlet of the system the mixing quality exceeds 95% exhibiting robust performance.

**Figure 8:**
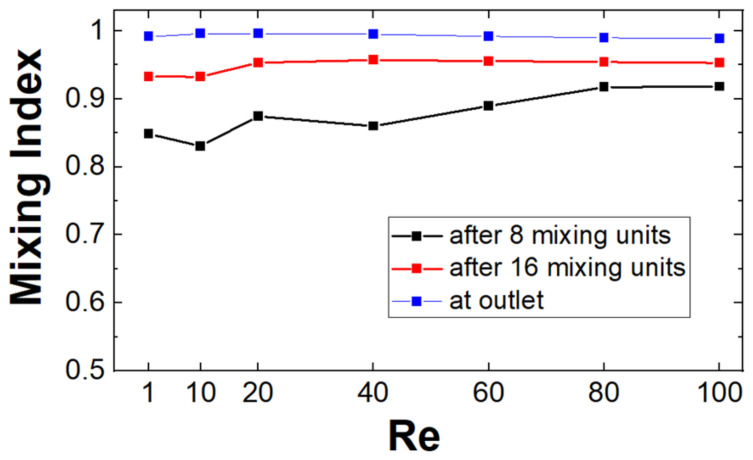
Performance as a function of the Reynolds number at different positions along the mixer for the optimized designs (blade twist angle = 130°, aspect ratio *AR* = 1, mobile phase fluid = water).

The performance of the proposed mixers is also consistent across a broad range of flow rates. The performance of mixers with two mixing units within the curvature is tested for mean inlet speeds ranging from 0.627 × 10^−3^ m·s^−1^ to 62.7 × 10^−3^ m·s^−1^ (*Re* = 1 to 100), corresponding to flow rates of 75 μL·min^−1^ to 7.5 mL·min^−1^. At intermediate positions, the achieved mixing quality increases with the flow rate, as the rotation imparted by the helical blades and the curvature of the channels to the fluid is dependent on the speed of the flow (Figure 8). By the outlet of all the mixers, though, the mixing performance is maximized for all the flow rates investigated with little variation with the Reynolds number of the system.

We have also analyzed the mixing in channels without mixing blades (i.e., in hollow pipes). For all the cases tested, the mixing achieved is much less than when the helical blades are present. As expected, the largest values are achieved in the topology with curvature pieces equivalent to two units. Even in those cases, the mixing indexes are 0.125 (after 8 equivalent units), 0.182 (after 16 equivalent units), and 0.2885 (at the outlet) for hollow pipes, whereas the indexes are 0.8305 (after 8 mixing units), 0.932 (after 16 mixing units), and 0.996 (at the outlet) in the optimized Kenics design (for *Re* = 10). Thus, while the Dean flows generated in the curved sections provide some mixing, this is significantly less than the effect of the mixing blades. This result is also consistent with the findings from the topology analysis, where topologies with sharper turns show slightly improved mixing performance.

**Figure 9:**
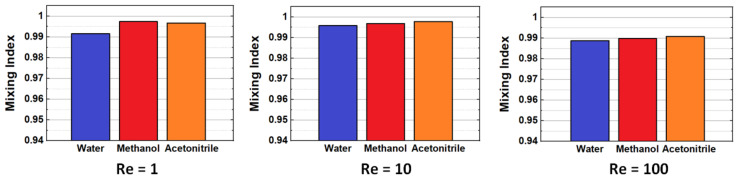
Mixing performance comparison at different Reynolds numbers for different working fluids, i.e., water, ethanol, and acetonitrile, respectively (blade twist angle = 130°, aspect ratio *AR* = 1).

Finally, the effect of the mobile phase fluid is also investigated. Simulations and mixing analysis are performed for mixers operating with three different solvents popular as mobile phases in LC: water, methanol, and acetonitrile, respectively, with physical properties as specified in Section 3. The mixing quality at the end of the mixers (24 mixing units) shows no statistically significant differences at all flow rates (Figure 9). Slightly larger values are obtained for acetonitrile and methanol relative to water, most probably because of their lower viscosity and higher diffusion constant, but for all conditions, the mixing performance is close to ideal, showing remarkable consistency across operating conditions. The mixer optimized here could have applications in fully integrated, miniaturized LC systems (e.g., chip technologies), where design flexibility along the longitudinal axis could enable efficient packing and portability without compromising the homogenization of fluids [35].

## 5. Conclusions

The development and successful optimization of a new inline mixer targeted toward enabling the mixing of mobile phases in liquid chromatography application has been presented. Inspired originally by the Kenics geometry popular for large-volume processing for industrial applications, the design is scaled down and adapted to volumes of the order of ~70 μL enabling robust mixing over the flow ranges from 75 μL·min^−1^ to 7.5 mL·min^−1^. Optimized designs, in terms of mixing blade twist angle and aspect ratio, exhibit excellent mixing performance over a broad range of operating conditions, fluids used, and even topology changes when assuming that the mixer is integrated within flexible capillary tubing. The latter opens the possibility that the primary mixing element, i.e., the periodic arrangement of twisted mixing blades, can be inserted in the existing tube connections of the system with no or minimum added volume. Future work will be directed towards the fabrication of the mixing elements accessible to rapid prototyping technologies such as 3D printing and implementation and testing in miniaturized LC systems.

## Data Availability

Not applicable.

## Supplementary Materials

The following supporting information can be downloaded at: https://www.mdpi.com/article/10.3390/mi14071373/s1. Supplementary Figure S1. (left) Velocity profiles across the channel for grids with a different number of elements as indicated in the legend; (right) Maximum velocity recorded across the profile as a function of the number of elements used in the discretization of the geometry. The mesh chosen for the simulations is highlighted by the blue circle (*Re* = 10; mobile phase fluid = water; blade twist angle = 130°; aspect ratio *AR* = 1).

## References

[1] Snyder L.R., Kirkland J.J., Dolan J.W. (2010). Introduction to Modern Liquid Chromatography.

[2] De Vos J., Broeckhoven K., Eeltink S. (2016). Advances in ultrahigh-pressure liquid chromatography technology and system design. Anal. Chem..

[3] Beccaria M., Cabooter D. (2020). Current developments in LC-MS for pharmaceutical analysis. Analyst.

[4] Langford J.B., Lurie I.S. (2022). Use of micro, capillary, and nano liquid chromatography for forensic analysis. J. Sep. Sci..

[5] Hussain C.M., Kecili R. (2020). Modern Environmental Analysis Techniques for Pollutants.

[6] Faraji M., Yamini Y., Gholami M. (2019). Recent advances and trends in applications of solid-phase extraction techniques in food and environmental analysis. Chromatographia.

[7] Žuvela P., Skoczylas M., Jay Liu J., Baczek T., Kaliszan R., Wong M.W., Buszewski B. (2019). Column characterization and selection systems in reversed-phase high-performance liquid chromatography. Chem. Rev..

[8] Stoll D. (2018). Mixing and Mixers in liquid chromatography—Why, When, and How Much? Part I, The Pump. LCGC N. Am..

[9] Kim K., Shah I., Ali M., Aziz S., Khalid M.A.U., Kim Y.S., Choi K.H. (2020). Experimental and numerical analysis of three Y-shaped split and recombination micromixers based on cantor fractal structures. Microsyst. Technol..

[10] Galletti C., Mariotti A., Siconolfi L., Mauri R., Brunazzi E. (2019). Numerical investigation of flow regimes in T-shaped micromixers: Benchmark between finite volume and spectral element methods. Can. J. Chem. Eng..

[11] Kromidas S. (2017). The HPLC Expert II: Find and Optimize the Benefits of Your HPLC/UHPLC.

[12] Samuelsson J., Shalliker R.A., Fornstedt T. (2017). Viscosity contrast effects in analytical scale chromatography-Evidence and impact. Microchem. J..

[13] Singh M.K., Anderson P.D., Meijer H.E. (2009). Understanding and optimizing the SMX static mixer. Macromol. Rapid Commun..

[14] Cocovi-Solberg D.J., Worsfold P.J., Miró M. (2018). Opportunities for 3D printed millifluidic platforms incorporating on-line sample handling and separation. TrAC—Trends Anal. Chem..

[15] Ianovska M.A., Mulder P.P.M., Verpoorte E. (2017). Development of small-volume, microfluidic chaotic mixers for future application in two-dimensional liquid chromatography. RSC Adv..

[16] Bian Y., Zheng R., Bayer F.P., Wong C., Chang Y.-C., Meng C., Zolg D.P., Reinecke M., Zecha J., Wiechmann S. (2020). Robust, reproducible and quantitative analysis of thousands of proteomes by micro-flow LC–ms/MS. Nat. Commun..

[17] Lenčo J., Vajrychová M., Pimková K., Prokšová M., Benková M., Klimentová J., Tambor V., Soukup O. (2018). Conventional-flow liquid chromatography–mass spectrometry for exploratory bottom-up proteomic analyses. Anal. Chem..

[18] Jet Weaver^TM^ Mixer (2011). Agilent 1290 Infinity Binary Pump Installation of the High Performance Jet Weaver.

[19] Paul E.L., Atiemo-Obeng V.A., Kresta S.M. (2019). Handbook of Industrial Mixing: Science and Practice.

[20] Otino J.M. (1989). The Kinematics Od Mixing: Stretching, Chaos, and Transport.

[21] Nguyen N.T. (2012). Micromixers: Fundamentals, Design and Fabrication.

[22] Fodor P.S., Vyhnalek B., Kaufman M. Entropic evaluation of Dean flow micromixers. Proceedings of the 2013 COMSOL Conference.

[23] Carson S.O., Covas J.A., Maia J.A. (2015). A New Extensional Mixing Element for Improved Dispersive Mixing in Twin-Screw Extrusion, Part 1: Design and Computational Validation. Adv. Polym. Technol..

[24] Tomaras G., Kothapalli C.R., Fodor P.S. (2022). Serpentine Micromixers Using Extensional Mixing Elements. Micromachines.

[25] Dortmund Data Bank. http://www.ddbst.com/online.html.

[26] Clark J.A., Butt T.A., Mahajan G., Kothapalli C.R., Kaufman M., Fodor P.S. (2019). Performance and implementation of centrifugal serpentine micromixers with non-rectangular cross-section. J. Micromech. Microeng..

[27] Jiang F., Drese K.S., Hardt S., Küpper M., Schönfeld F. (2004). Helical flows and chaotic mixing in curved micro channels. AIChE J..

[28] Meijer H.E.H., Singh M.K., Anderson P.D. (2012). On the performance of static mixers: A quantitative comparison. Prog. Polym. Sci..

[29] Galaktiomov O.S., Anderson P.D., Peters G.W.M., Meijer H.E.H. (2003). Analysis and optimization of Kenics static mixers. Int. Polym. Process..

[30] van Wageningen W.F., Kandhai D., Mudde R.F., van den Akker H.E. (2004). Dynamic flow in a Kenics static mixer: An assessment of various CFD methods. AIChE J..

[31] Juraeva M., Kang D.J. (2021). Optimal combination of mixing units using the design of experiments method. Micromachines.

[32] Ortega-Casanova J. (2016). Enhancing mixing at very low Reynolds number by a heaving square cylinder. J. Fluids Struct..

[33] Jiang X., Yang N., Wang R. (2021). Effect of aspect ratio on the mixing performance in the Kenics static mixer. Processes.

[34] Khaydarov V., Borovinskaya E.S., Reschetilowski W. (2018). Numerical and experimental investigations of a micromixer with chicane mixing geometry. Appl. Sci..

[35] Medina D.A.V., Maciel E.V.S., Lancas F.M. (2020). Miniaturization of liquid chromatography coupled to mass spectrometry. 3. Achievements on chip-based LC–MS devices. TrAC—Trends Anal. Chem..

