# Supplementary material for: A Flexible Kenics Mixer for Applications in Liquid Chromatography
Source: Micromachines (Basel). 2023 Jul 4;14(7):1373. doi: 10.3390/mi14071373 (PMC10386428; doi:10.3390/mi14071373)
Supplement: Supplementary file 1 [file micromachines-14-01373-s001.zip › micromachines-2448109-supplementary.pdf]

Supplementary Information

Journal: Micromachines (ISSN 2072-666X)

Type: Article

Title: A Flexible Kenics Mixer for Applications in Liquid Chromatography

Authors: Prachet DSK, Petru S Fodor*, Chandrasekhar R Kothapalli*

We have chosen for the simulations grids that seek to minimize numerical errors while limiting the computational expense. The grid analysis is shown in Supplementary Figure 1, below. Here, the velocity profiles obtained for simulations with different mesh resolutions are compared. The profiles are collected across the stream as it passes through one of the mixing elements (specifically mixing unit 9). All profiles show two maxima corresponding to the semicircular channels defined by the helical blade. For rough grids (low number of mesh elements) the velocity profiles show artificial roughness related with the low grid resolution, as well as non-zero values in the volume occupied by the mixing blade. As the mesh finesse is increased the quality of the results improves dramatically, with profiles obtained for high resolution grids overlapping very well both in the center of the channels, as well as close to the walls and the mixing blade. As highlighted in Figure 1 below, we have chosen for the simulations, grids with more than 3.5 million elements. For the grids chosen the accuracy for the velocities is within better than 1% of those from simulations with much higher grid resolutions, while keeping the models computationally efficient.

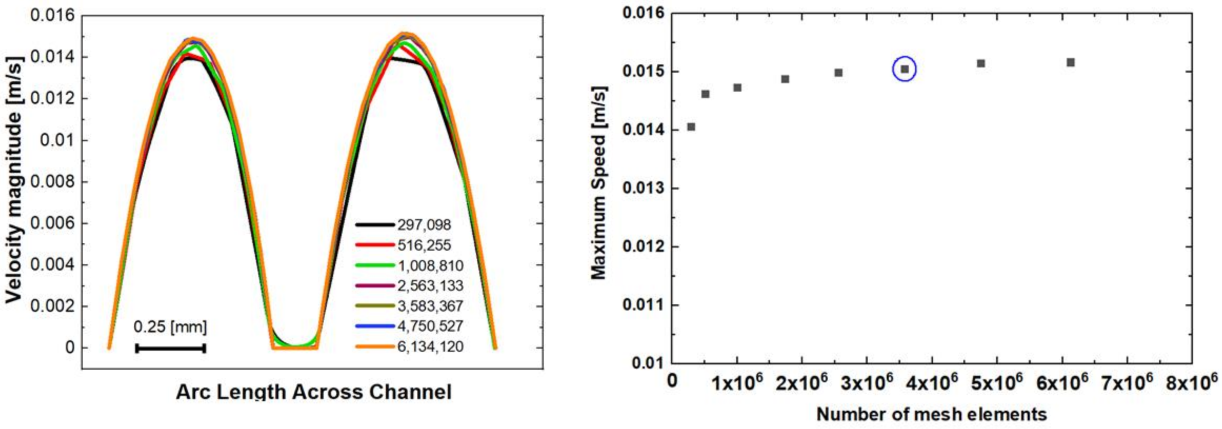

Figure S1. (left) Velocity profiles across the channel for grids with different number of elements as indicated in the legend; (right) Maximum velocity recorded across the profile, as a function of the number of elements used in the discretization of the geometry. The mesh chosen for the simulations is highlighted by the blue circle (Re = 10; mobile phase fluid = water; blade twist angle = 130°; aspect ratio AR = 1).
